# Nutritional knowledge and eating habits of professional rugby league players: does knowledge translate into practice?

**DOI:** 10.1186/s12970-015-0082-y

**Published:** 2015-04-17

**Authors:** Ieva Alaunyte, John L Perry, Tony Aubrey

**Affiliations:** Leeds Trinity University, Department of Sport, Health and Nutrition, Brownberrie Lane, Horsforth, LS18 5HD UK; Liverpool Hope University, School of Health Sciences, Hope Park, Liverpool, L16 9JD UK

**Keywords:** Diet, Elite, Rugby league, Athletes, Sport

## Abstract

**Background:**

Adequate nutrient intake is important to support training and to optimise performance of elite athletes. Nutritional knowledge has been shown to play an important role in adopting optimal nutrition practices. The aim of the present study was to investigate the relationship between the level of nutritional knowledge and dietary habits in elite English rugby league players using the eatwell plate food categories.

**Method:**

General nutritional knowledge questionnaires were collected during the Super League competitive season in the first team squad of 21 professional Rugby league players (mean age 25 ± 5 yrs, BMI 27 ± 2.4 kg/m2, experience in game 6 ± 4 yrs). According to their nutritional knowledge scores, the players were assigned to either good or poor nutritional knowledge group (n = 11, n = 10, respectively). Their dietary habits were assessment using a food frequency questionnaire.

**Results:**

The findings revealed that nutritional knowledge was adequate (mean 72.82%) in this group of athletes with the highest scores in dietary advice section (85.71%), followed by food groups (71.24%) and food choice (69.52%). The majority of athletes were not aware of current carbohydrate recommendations. This translated into their dietary habits as many starchy and fibrous foods were consumed only occasionally by poor nutritional knowledge group. In terms of their eating habits, the good nutritional knowledge group consumed significantly more fruit and vegetables, and starchy foods (p <.05). Nutritional knowledge was positively correlated to fruit and vegetables consumption (rs = .52, p <.05) but not to any other eatwell plate categories.

**Conclusions:**

The study identified adequate general nutritional knowledge in professional rugby league players with the exception of recommendation for starchy and fibrous foods. Players who scored higher in nutritional knowledge test were more likely to consume more fruits, vegetables and carbohydrate-rich foods.

## Background

Rugby League is a physically demanding sport that is characterised by repeated changes in exercise intensity, from low-speed activity such as standing and walking to high-intensity bouts such as sprints and tackles [1]. These physiological demands place heavy requirements on body’s fuel stores. An adequate nutritional intake is essential for promoting overall health as well as for optimal training and competition performance of professional rugby league players.

Despite the increased interest in nutrition amongst many elite athletes and the well documented importance of a balanced diet for overall health and athletic performance, research suggests that professional rugby players consume diets that are less than optimal [2-4]. Furthermore, nutritional knowledge of elite athletes and their coaches was shown to be inadequate [5-7]. It is argued that nutritional knowledge is strongly associated with healthy eating and healthy food choices irrespective of demographic variables [8]. In addition to this, Rugby League players generally tend to focus much less on their diet compared to endurance athletes [9], which coupled with inadequate nutritional knowledge may result in poorer dietary choices.

The basis of adequate nutrient intake is a healthy and balanced diet, therefore, recommendations for athletes is to consume a diet, which follows current dietary guidelines for general population. The eatwell plate is used by health professionals as the national recommendation for healthy diets in the UK [10]. The main nutritional aim of the eatwell plate is to help individuals to understand the relative proportions of different food groups in a healthy and well balanced diet. Healthy adults are encouraged to consume at least five portion of fruit and vegetables a day, plenty of bread, rice, potatoes, pasta and other starchy foods (choosing wholegrain varieties where possible), some milk and dairy foods, some meat, fish (two portions a week, one of which should be oily), eggs, beans and other non-dairy sources of protein and just a small amount of foods and drinks high in fat and/or sugar [10]. The eatwell plate does not provide guidance to frequency of serving and portion sizes (with the exception of fruit, vegetables and fish) because it was developed as a tool suitable for most adults, hence, it would be misleading to include specific portion advice. Nevertheless, the eatwell plate provides food group daily proportions (starchy foods- 33%, fruit and vegetables- 33%, milk and dairy foods- 15%, meat, fish, eggs, beans and other non-dairy sources of protein- 12%, foods and drinks high in fat and/or sugar- 8%) and can be used by registered dietitians and nutritionists in giving individually tailored advice.

A number of previous studies evaluated the adequacy of the diets of elite rugby league players by comparing individual nutrients to current recommendations [3,4]. However, there is a lack of literature evaluating eating habits and diets of elite athletes using a food group analysis approach.

The aim of this study was to determine general nutritional knowledge and eating habits based on the eatwell plate food groups’ categories of elite rugby league players, and to explore any associations between good or poor nutritional knowledge and eating habits in this population.

## Methods

### Participants

Thirty professional male Rugby League players were recruited from one team competing in English Super League. All athletes were part of the first team squad. The data was collected during the 2012–13 competition season.

The study was approved by the Ethics Committee of a UK Higher Education institution. Written consent was collected from all participants.

Table 1 summarises study subject characteristics. The age of participants ranged from 18 to 34 years with an average age of 25. All participants had educational qualifications with the majority holding GSCEs (38%), followed by A-levels (19%) and diplomas (19%). None of the participants had any special dietary needs or were on any special diet at the time of the investigation.

**Table 1:** **Characteristics of participants**

**Characteristics**	**Participants**
n	21
Age (y)	25 (*SD* = 5)
Height (m)	1.8 (*SD* = 0.1)
Weight (kg)	93 (*SD* = 12)
Game experience (years in Super League)	6 (*SD* = 4)
**Position played (n):**	
Back	9
Forward	12
**Level of education (n):**	
Secondary	3
GCSE	8
A- levels	4
Diploma	4
Degree	2
Health and/or nutrition related qualifications (n)	1
Special dietary needs/conditions (n)	0

### Measures and procedure

Subject characteristics including age, position played, game experience, level of education, health or nutrition related qualifications and any current special dietary regimes were collected using a short questionnaire.

Nutritional knowledge questionnaire (NKQ) [11] consisted of 28 questions categorised into three sections:

section A – advice - current national recommendations and heath experts’ advice on nutrition;
section B – food groups - classification of food items into the food groups, including foods low and high in sugar, fat, salt, protein, fibre;
section C – food choice - choosing healthier food options

Food frequency questionnaire (FFQ) [12] was chosen due to the availability of local food items to account for region-specific dietary habits and consisted of 78 food and beverage items (including examples of standard portion sizes for each item), categorised into seven sections: starchy foods (breads and crackers, breakfast cereals, potatoes, rice and pasta), protein-rich foods (meat, poultry, fish, eggs and beans), dairy products (milk, yoghurt, cheese), vegetables (fresh, frozen, canned), fruits (fresh, frozen, canned, dried), foods high in sugar or/and fat, and fluids (water, tea, coffee, fresh fruit juice, juice drinks, quash, low calorie drinks and fizzy drinks).

The questionnaires were distributed to participants prior a training session and collected straight after completion.

### Data analysis

Preliminary analysis screened the data for missing values, outliers, and univariate normality. Data was screened for missing values, of which there were less than 0.1% and examined for outliers using Q-Q plots. No problematic outliers were found. Most food categories demonstrated no issue with skewness (<2), though a slight negative skew was evident for starchy foods (−2.80), fluids (−2.20), and sugary foods (−2.04). All kurtosis statistics were acceptable (<7).

Nutritional knowledge was calculated as the summed total of correct answers from the NKQ, including sub-dimensions of expert advice, food groups, and food choice. Relationships between the subsections of the NKQ and food frequency were examined by Spearman’s rank order. High and low nutritional knowledge groups were identified by a median split and a chi-square difference test was conducted to examine group differences. Similarly, we then examined the relationship between nutritional knowledge and the eatwell plate groups of starchy foods, protein, fruit and vegetables, milk and dairy, fats, and fluids.

## Results

### Nutritional knowledge

A response rate of 70% (*n* = 21) was achieved due to exclusion of partially completed NKQ and FFQ. The study sample comprised of male rugby league athletes (nine backs and 12 forwards) with an average professional competition experience of six years.

The average number of nutritional knowledge questions answered correctly was 52.43 (*SD* = 4.40), representing a mean nutritional knowledge score of 72.82% (*SD* = 6.11). The minimum score was 62.50% and the maximum score was 83.33%. Questions related to *advice* were answered most accurately (*M* = 85.71%, *SD* = 13.04). Questions pertaining to food groups (*M* = 71.24%, *SD* = 7.21) and food choice (*M* = 69.52%, *SD* = 13.96) were answered less accurately. To further investigate nutritional knowledge, the sample was split into two groups relative to the median NFQ score. This equated to a sample of 10 players in the *poor nutritional knowledge (NK)* group and 11 players in the *good nutritional knowledge (NK)* group. These results are presented in Table 2. Overall, the good nutritional knowledge group scored significantly higher in *food groups* (76.29%) than the poor nutritional knowledge group (65.69%; *t*(19) = 4.98, *p* <.01). They also scored significantly higher in *advice* (92.56% to 78.18%; *t*(19) = 2.98, *p* <.01), and overall (77.78% to 67.36%; *t*(19) = 7.78, *p* <.01). No significant difference was observed for *choice* (73.64% to 64.00%; *t*(19) = 1.46, *p* = .16).

**Table 2:** **Nutritional knowledge scores**

**Nutrition knowledge categories:**	**ALL (n = 21)**		**Good NK group (n = 11)**		**Poor NK group (n = 10)**		***p*** **value (between groups)**
	**Mean (SD)**	**%**	**Mean (SD)**	**%**	**Mean (SD)**	**%**	
A: Dietary advice (11 points)	9.43 (1.43)	85.71	10.18 (.26)	92.56	8.60 (.48)	78.18	0.01
B: Food groups (51 points)	36.33 (3.68)	71.24	38.91 (.55)	76.29	33.50 (.97)	65.69	0.01
C: Food choice (10 points)	6.95 (1.40)	69.52	7.36 (.39)	73.64	6.50 (.45)	64.00	0.16
Overall score (72 points)	52.43 (4.4)	72.82	56.00 (.69)	77.78	48.50 (.67)	67.36	0.01

Table 3 shows comparisons of the frequency of consumption of food items of different food groups between good and poor nutritional knowledge groups. Starchy foods, fruit & vegetables, oily fish and milk were consumed more frequently by good nutritional knowledge group when compared to poor nutritional knowledge group. Furthermore, the latter reported more frequent consumption of fizzy drinks and squash compared to the good nutritional knowledge group. There was less variation in consumption of meat and poultry products, eggs, foods that are high in sugar and/or fat and other beverages.

**Table 3:** **Descriptive statistics and between group comparisons for food frequency**

**Food group**	**Good Nutritional Knowledge**			**Poor Nutritional Knowledge**		
	**Never/Rarely**	**Occasionally**	**Often**	**Never/Rarely**	**Occasionally**	**Often**
**Starchy foods:**						
White bread	72.7%	18.2%	9.1%	60%	30%	10%
Brown bread	18.2%	36.4%	45.5%	20%	40%	40%
Breakfast Cereals	0%	18.2%	81.8%	10%	30%	60%
Boiled and baked potatoes	18.2%	81.8%	0%	60%	40%	0%
Chips	90.9%	9.1%	0%	60%	40%	0%
Rice	72.7%	27.3%	0%	60%	40%	0%
Pasta	9.1%	36.4%	54.5%	10%	70%	20%
**Protein-rich foods:**						
Grilled/ baked meat or poultry	36.4%	63.6%	0%	50%	40%	10%
Meat casseroles or stews	27.3%	72.7%	0%	70%	30%	0%
White fish	90.9%	9.1%	0%	80%	20%	0%
Oily fish	0%	81.8%	18.2%	30%	60%	10%
Beans and pulses	36.4%	36.4%	27.3%	60%	30%	10%
Eggs	0%	81.8%	18.2%	20%	60%	20%
**Fruits**	18.2%	81.8%	0.0%	40.0%	60.0%	0.0%
**Vegetables**	9.1%	90.9%	0.0%	50.0%	50.0%	0.0%
**Milk and dairy products:**						
Milk	72.7%	0%	27.3%	100%	0%	0%
Yoghurt	18.2%	54.5%	27.3%	40%	20%	40%
Cheese	18.2%	81.8%	0%	10%	60%	30%
**Foods high in fats and/or sugar:**						
Chocolate	81.8%	18.2%	0%	70%	30%	0%
Crisps	90.9%	0%	9.1%	60%	40%	0%
Jam	63.6%	27.3%	9.1%	80%	10%	10%
Margarine	9.1%	18.2%	72.7%	40%	20%	40%
**Fluids:**						
Water	0%	9.1%	90.9%	0%	0%	100%
Fresh fruit juice	9.1%	36.4%	54.5%	20%	50%	30%
Juice drinks	45.5%	36.4%	18.2%	50%	20%	30%
Fizzy drinks	90.9%	9.1%	0%	40%	20%	40%
Squash	81.8%	18.2%	0%	70%	20%	10%
Low calorie drink	81.8%	9.1%	9.1%	80%	10%	10%
Tea	36.4%	36.4%	27.3%	40%	20%	40%

### Nutritional knowledge and food frequency

Spearman’s rank order correlations were calculated to first examine the relationship between nutritional knowledge and food consumption. Relationships among variables were generally weak, with the exception of *advice* score, which correlated significantly with vegetable consumption (*r*_*s*_ = .49, *p* < .001).

Cross-tabulated chi-square difference tests found that the good nutritional knowledge group consumed significantly more vegetables (*p* < .05). Specifically, the good nutritional knowledge group consumed significantly (*p* < .05) more breakfast cereal, boiled/baked potatoes, meat casseroles/stews, peas/green beans, broccoli, cabbage and spring greens, cauliflower, peaches, nectarines and melons, strawberries, raspberries, mango and kiwi. In no food types did the poor nutritional knowledge group consume significantly more than the good nutritional knowledge group.

### The eatwell plate analysis

Spearman’s rank correlations demonstrated weak relationship between nutritional knowledge and food consumption in relation to the *eatwell* plate (Table 4). The only statistically significant relationship was the positive correlation between fruit and vegetable consumption and overall nutritional knowledge (*r*_*s*_ = .52, *p* <.05). Differences between nutritional knowledge groups were assessed through an independent samples t test, which supported the effect for fruit and vegetables (*t*(19) = 3.31, *p* <.01), which was significantly higher in the good nutritional knowledge group (*M* = 30.91, *SD* = 3.08) than the poor nutritional knowledge group (*M* = 24.60, *SD* = 5.44). Inferential statistical analysis could not detect any significant differences among other *eatwell* plate categories.

**Table 4:** **Descriptive statistics and correlations of food consumption with nutritional knowledge**

**Food Group**	**Mean frequency score**	**SD**	**Skew**	**Kurt**	**Advice**	**Food groups**	**Choice**	**Overall**
Starchy	28.00	5.21	−2.30	5.31	.15	-.02	.11	.13
Protein-rich	14.81	1.40	.86	3.51	-.01	-.10	.01	-.03
Fruit and Vegetables	27.90	5.34	-.62	-.49	.38	.36	.33	.52*
Milk and Dairy	7.43	1.47	.10	-.71	.06	.18	.12	.18
Fats/Sugars	16.86	1.62	.80	1.40	.22	.02	-.12	.11
Fluids	11.52	1.57	1.67	2.96	-.16	-.28	-.34	-.29

*Statistically significant at *p* <.05.

## Discussion

The present study aimed to determine nutritional knowledge and eating habits based on the national healthy eating recommendations tool (the eatwell plate) categories, and to evaluate the relationship between nutritional knowledge and dietary practices in elite rugby league athletes.

The mean nutritional knowledge score was 73% for elite athletes in the present study. This is higher compared to the findings observed in the population of elite Australian rugby league athletes [7]. The authors reported the mean score of 61.3% using an adapted 90-point nutritional knowledge questionnaire whilst the present study used 72-points of the same questionnaire; therefore, caution should be taken when making direct comparisons. Nevertheless, differences in nutritional knowledge scores between the studies could be explained by several factors. Firstly, athletes in the present study were notably older (25 compared to 19 years of age) and obtained higher level of education (100% of athletes had secondary and above education in the present study compared to 66% in the Australian athletes). Both variables, age and the level of education, have been previously shown to be factors in influencing nutritional knowledge [8].

Knowledge of dietary recommendations section was the most accurately completed (86% accurate answers) compared to other sections (food groups – 71%, food choice – 70%). When sample was split into good and poor nutritional knowledge groups, the trend remained the same. Consistent with the findings of another study [7], this suggests that the national health promotion campaigns, such as 5-a-day fruit and vegetables, salt reduction, and increased wholegrain intake, are successful educational public health nutrition programmes, in terms of raising public awareness and increasing nutritional knowledge. In fact, all of the participants completed the question on recommended servings of daily fruit and vegetables intake correctly.

Despite the international sports nutrition recommendations [13] and the national healthy eating guidelines [10] being based on moderate consumption of starchy staple foods, the athletes in the present study were unaware of these recommendations. All participants in the poor NK group and nearly half in the good NK group answered the questions on starchy foods consumption incorrectly (‘Do you think health experts recommended that people should be eating more, the same amount, or less of starchy foods’, ‘Do you think experts put these in the starchy foods group?’). One plausible explanation would be the current public perception of carbohydrate-rich foods being unhealthy and in fact the majority of dietary advice provided by popular media incorporates this carbohydrate recommendation poorly [14]. This trend also translated into the eating habits of the elite athletes observed in the present study. The majority of rugby players consumed starchy carbohydrate-rich foods ‘occasionally’, with the exception of breakfast cereals which were reported to be consumed ‘often’. Further to this, the poor NK group reported to consume significantly less starchy foods, including boiled and baked potatoes and shredded wheat breakfast cereals, compared to good NK group. It is well acknowledged that in the athlete’s diet the majority of energy should come from carbohydrates as they are used as a predominant source of fuel during an intense and prolonged exercise [13]. In addition to this, a recent study highlighted inadequate carbohydrate intake in collegiate rugby players [2]. Therefore, there is a need for public health initiatives outlining the important of adequate consumption of carbohydrate-rich foods in physically active populations.

The good NK group had significantly better knowledge of high fibre foods in relation to the amount they should be consuming compared to the poor NK group. This was also reflected in their dietary practices. The good NK group included significantly more high fibre foods, such as breakfast cereals, potatoes, peas, green beans, broccoli and cauliflower, cabbage and spring greens, peaches and nectarines, melons, strawberries, raspberries, mango and kiwi, in their diets. In addition to this, the eatwell plate analysis showed the positive association between overall nutritional knowledge and consumption of fruit and vegetables (rs = .52, p < .05). Similar observations were reported in the general UK population study comprising 455 men and 584 women aged 18 to 65 years and over [8]. The highest quintile for nutritional knowledge was 25 time more likely to meet fruit and vegetables recommended daily intake than those individuals in the lowest quintile. In addition, the association between nutritional knowledge and fruit and vegetables intake was independent to gender, education and occupational class. This suggests that associations observed in the present study of elite male rugby league players follow similar patterns to the findings of general population.

Overall, there was a lack of relationship between the eatwell plate food categories and nutritional knowledge. Although a relative small sample size may explain the lack of significant associations, it may be suggested that the adequate nutritional knowledge observed may not have translated into the appropriate dietary practice in present study’s cohort. Only a weak (rs <.44) positive correlation between nutritional knowledge of athletes and dietary intake was reported in five out of nine studies included in a recent systematic review [15]. In addition to this, the concept of nutritional knowledge encompasses two elements: knowledge of nutrition facts and procedural knowledge, such as planning, purchasing and preparation of foods [16]. Therefore, if declarative knowledge is adequate but procedural knowledge is poor, it is unlikely that nutritional knowledge would play a big role in the adoption of healthier food habits. Literature also indicates that female participants and athletes involved in sports with a strong emphasis on physique and those participating in endurance sports have better knowledge compared to male athletes and inactive controls [17-19]. If this is the reason for the lack of association between good knowledge and dietary practices in the cohort of male rugby league players of the present study, the future nutrition education programmes for athletes should target to improve general nutritional knowledge through incorporating procedural approaches in this population.

## Conclusions

The present study assessed the level of general nutritional knowledge and eating habits of elite rugby league players.

Nutritional knowledge was adequate for dietary advice section but poorer for food groups classification and food choice sections. Further analysis of the present study suggests that the knowledge could be improved in relation to the current recommendations of increasing the consumption of carbohydrate-rich foods.

There was a lack of association observed in terms of other eatwell plate food groups and nutritional knowledge, which is most likely to be influenced by a relatively small sample size. Nevertheless, a link between knowledge and fruit and vegetables consumption was identified, which suggests that improving other domains of nutritional knowledge may be beneficial in improving the overall diet in this population.

The present study highlights the importance of qualified nutritional support at the elite level of rugby. It is recommended to appraise at the barriers of nutritional knowledge in both elite athletes and their coaches to understand why adequate nutritional knowledge may not always result in better nutrition practices.

### Availability of supporting data

All the data from this research are available on your request.
